# Our graft choice for surgical treatment of true aneurysms of subclavian and axillary arteries

**DOI:** 10.1186/1749-8090-8-S1-P48

**Published:** 2013-09-11

**Authors:** O Gokalp, I Yurekli, L Yilik, U Yetkin, T Gunes, M Akyuz, B Ozcem, O Tetik, G Ilhan, A Gurbuz

**Affiliations:** 1 Department of Cardiovascular Surgery, Izmir Katip Celebi University Ataturk Training and Research Hospital, Izmir, Turkey; 2 Rize University Medical Faculty Department of Cardiovascular Surgery, Rize, Turkey

## Background

Both subclavian and axillary artery aneurysms should be promptly treated after diagnosis due to complications because the possibility of the aneurysm to rupture or to thrombose increases when the diameter of the aneurysm increases. Similarly, the possibility of aneurysm-related embolization and development of neurological complication increases.

## Methods

Eight patients that were operated on between February 1998 and December 2007 due to true aneurysms of the subclavian and axillary arteries were examined. Six of the patients (75%) were male. Median age was 58 (38-73). Two of these patients had subclavian and 6 of them had axillary artery aneurysms.

## Results

One patient with subclavian artery aneurysm underwent resection of the aneurysm and subclavian-axillary bypass surgery with brachial embolectomy using a polytetrafluoroethylene (PTFE) graft; whereas the other patient underwent resection of the aneurysm and subclavian-axillary bypass surgery with saphenous vein graft. In addition to resection of the aneurysm, three out of 6 patients with axillary artery aneurysm underwent saphenous vein graft interposition and remaining 3 underwent PTFE graft interposition. Moreover, one patient with acute ischemia underwent an additional brachial embolectomy.

## Conclusions

The surgical treatment of the axillary and subclavian artery aneurysms usually consists of resection of the aneurysm and interposition of a graft. Saphenous vein graft, PTFE or Dacron grafts are usually used as conduits. The established practice is choosing saphenous vein whenever possible. If saphenous vein is not available due to low quality, diameter mismatch or prior use of saphenous vein conduit (coronary bypass, etc…); then synthetic graft material is used. We used saphenous vein in 4 patients and PTFE graft in remaining 4.

